# Extracellular ATP targets Arabidopsis RIBONUCLEASE 1 to suppress mycotoxin stress‐induced cell death

**DOI:** 10.1111/nph.18211

**Published:** 2022-05-31

**Authors:** Heather L. Goodman, Johan T. M. Kroon, Daniel F. A. Tomé, John M. U. Hamilton, Ali O. Alqarni, Stephen Chivasa

**Affiliations:** ^1^ Department of Biosciences Durham University South Road Durham DH1 3LE UK

**Keywords:** Arabidopsis, extracellular ATP, fumonisin B1, programmed cell death, RNS1, salicylic acid, *S*‐like ribonuclease

## Abstract

- Extracellular ATP is a purinergic signal with important functions in regulating plant growth and stress‐adaptive responses, including programmed cell death. While signalling events proximate to receptor activation at the plasma membrane have been characterised, downstream protein targets and the mechanism of cell death activation/regulation are unknown.
- We designed a proteomic screen to identify ATP‐responsive proteins in Arabidopsis cell cultures exposed to mycotoxin stress via fumonisin B1 (FB1) application.
- Arabidopsis RIBONUCLEASE 1 (RNS1) was identified by the screen, and transgenic plants overexpressing native RNS1 showed greater susceptibility to FB1, while a gene knockout *rns1* mutant and antisense *RNS1* transgenic plants were resistant to FB1‐induced cell death. Native RNS1 complemented *rns1* mutants and restored the cell death response to FB1, while a catalytically inactive version of the ribonuclease could not. The FB1 resistance of salicylic acid (SA)‐depleted *nahG*‐expressing plants was abolished by transformation with native RNS1, but not the catalytically dead version.
- The mechanism of FB1‐induced cell death is activation of RNS1‐dependent RNA cleavage, which is blocked by ATP via *RNS1* suppression, or enhanced by SA through induction of *RNS1* expression. Our study reveals RNS1 as a previously unknown convergence point of ATP and SA signalling in the regulation of stress‐induced cell death.

## Introduction

Plants have an intracellular pool of ATP used to provide energy for metabolic reactions, and an extracellular pool of ATP used for cell signalling. Extracellular ATP (eATP) signalling has important roles in plants, regulating diverse physiological processes and stress‐adaptive responses. General plant growth (Wu *et al*., 2007; Riewe *et al*., 2008; Clark *et al*., 2010; Tonón *et al*., 2010) and the development and function of specific cell types and organs is regulated by eATP. For example, eATP drives pollen germination and pollen tube expansion (Steinebrunner *et al*., 2003; Reichler *et al*., 2009), root gravitropic growth (Tang *et al*., 2003) and root tuber (Riewe *et al*., 2008) and nodule (McAlvin & Stacey, 2005; Tanaka *et al*., 2011) formation, and control of stomatal guard cells (Clark *et al*., 2011; Hao *et al*., 2012; Chen *et al*., 2017). Furthermore, eATP regulates adaptive responses to biotic stress (Chivasa *et al*., 2005; Chen *et al*., 2017; Tripathi *et al*., 2018) and abiotic stress (Thomas *et al*., 2000; Kim *et al*., 2009; Sun *et al*., 2010; Hou *et al*., 2018). The diversity of developmental and stress‐adaptive processes controlled by eATP highlights its fundamental importance in plants. However, the mechanisms of eATP functions are not yet fully understood.

Extracellular ATP signals through plasma membrane receptors. The first receptor of this type to be characterised in plants was the Arabidopsis DOES NOT RESPOND TO NUCLEOTIDES 1 (DORN1), also named P2K1. The second Arabidopsis ATP receptor, P2K2, has been proposed to form a receptor complex with P2K1, with the latter transphosphorylating the former when the co‐receptors bind ATP on their extracellular domains (Pham *et al*., 2020). ATP binds DORN1/P2K1 or P2K2 to activate a surge in cytosolic calcium (Ca^2+^) concentration (Choi *et al*., 2014; Pham *et al*., 2020). In Arabidopsis, ANNEXIN 1 (Mohammad‐Sidik *et al*., 2021), CYCLIC NUCLEOTIDE‐GATED ION CHANNEL 2 (CNGC2) (Wu *et al*., 2021; Wang *et al*., 2022), CNGC4 (Wu *et al*., 2021), and CNGC6 (Duong *et al*., 2021) are plasma membrane ion channels gating Ca^2+^ influx in response to receptor activation, though the mechanistic link between the ion channels and ATP receptors is still unclear. Recently, mevalonate kinase (MVK) was identified as a critical component in ATP‐induced Ca^2+^ influx. MVK directly interacts with and is transphosphorylated by DORN1/P2K1 in response to ATP, with loss‐of‐function *mvk* mutants losing the ability to recruit calcium signalling in response to eATP (Cho *et al*., 2022). Whether MVK is between DORN1/P2K1 and the calcium channels is not yet clear.

In addition to the distinctive biphasic ATP‐induced Ca^2+^ signature (Matthus *et al*., 2019), receptor activation triggers rapid biosynthesis of other second‐messengers, such as nitric oxide (NO) (Foresi *et al*., 2007; Wu & Wu, 2008; Clark *et al*., 2010), reactive oxygen species (Song *et al*., 2006; Wu *et al*., 2008; Demidchik *et al*., 2009; Chen *et al*., 2017), and phosphatidic acid (Sueldo *et al*., 2010). These early signalling events activate changes in gene expression which underpin ATP regulation of diverse physiological processes.

*DORN1*‐overexpressing plants or loss‐of‐function *dorn1* mutants enabled the identification of gene networks downstream of eATP. Choi *et al*. (2014) used transcriptomics to identify genes responsive to ATP in a DORN1‐dependent manner, although they were not assigned to specific physiological processes controlled by ATP. Jasmonate‐dependent reprogramming of the pathogen defence transcriptome activated by ATP was revealed by Tripathi *et al*. (2018), with the influence of salicylic acid (SA) and ethylene signalling on shaping the transcriptome reported in a later study from the Tanaka laboratory (Jewell *et al*., 2019). Altering eATP concentration by application of the glucose‐hexokinase ATP‐trap (Chivasa *et al*., 2005) or exogenous application of a nonhydrolysable ATP analogue (Chivasa *et al*., 2010) identified protein networks responsive to the depletion, or increased concentration, of eATP. Genetic suppression of Arabidopsis apyrases (ATP‐hydrolases) increased the steady‐state eATP concentration, leading to identification of ATP‐responsive genes encoding growth‐regulatory cell wall proteins and stress‐responsive proteins (Lim *et al*., 2014). Nevertheless, the mechanisms by which transcriptionally regulated downstream genes/proteins function in specific physiological processes are currently unknown – this information is critical to understanding how eATP works in plants.

We have a longstanding interest in the ATP‐responsive proteins underpinning its cell death regulatory functions. Our strategy is to identify proteins that respond to ATP under stress using the cell death‐inducing mycotoxin fumonisin B1 (FB1). FB1 inhibits ceramide synthase and disrupts sphingolipid biosynthesis (Merrill *et al*., 1993). Loss‐of‐function mutation of genes encoding subunits of serine palmitoyltransferase, the enzyme catalysing the commitment step in sphingolipid biosynthesis, confers resistance to FB1 (Shi *et al*., 2007; Saucedo‐García *et al*., 2011), providing genetic evidence that sphingolipids are essential for cell viability. However, while exogenous ceramide can rescue animal cells from FB1‐induced programmed cell death (PCD) (Harel & Futerman, 1993), it fails to prevent cell death in Arabidopsis (Stone *et al*., 2000), showing that additional factors unrelated to ceramide depletion are involved in the Arabidopsis cell death response. Accordingly, we found that FB1 triggers eATP depletion before activation of cell death, and that exogenous ATP blocks Arabidopsis cell death (Chivasa *et al*., 2005). Furthermore, we recently demonstrated that the secreted ATP‐binding PHOSPHOLIPASE‐LIKE1 protein is essential for FB1‐induced cell death (Smith *et al*., 2021). Here, we designed a screen to identify proteins responding to ATP in FB1‐stressed Arabidopsis cells and identified RIBONUCLEASE 1 (RNS1) as a key target protein for ATP regulation of cell death. We show that the mechanism of cell death activation by RNS1 is through RNA degradation, a strategy similarly used by *S*‐locus ribonucleases in killing self‐pollen to prevent inbreeding. ATP suppresses FB1‐induced cell death by downregulating *RNS1* expression.

## Materials and Methods

### Plant material and chemicals

*Arabidopsis thaliana* (L.) Heynh. cell suspension cultures were grown under a 16 h : 8 h, light : dark photoperiod as described previously (Chivasa *et al*., 2005). Cells were used for experiments 3 d after sub‐culturing. Arabidopsis plants were grown in soil at 22°C under a 16 h : 8 h, light : dark photoperiod (*c*. 70 µmoles m^−2^ s^−1^) and were used for experiments 4–6 wk later. Transgenic Arabidopsis plants expressing the bacterial salicylate hydroxylase *nahG* gene in the Col‐0 ecotype were used. All T‐DNA insertion lines were in the Col‐0 ecotype and were obtained from the SALK collection (Alonso *et al*., 2003). Both *plcl1‐1* (SALK_048688) and *plcl1‐2* (SALK_023867) have impaired *PLCL1* (At1g13680) expression (Smith *et al*., 2021), while SALK_087165.56.00.X (referred to hereinafter as *rns1*) has a T‐DNA insertion in the promoter region of *RNS1*. An antisense *RNS1* (*asRNS1*) transgenic line (Bariola *et al*., 1999) was kindly donated by Professor Gustavo MacIntosh. FB1 from *Fusarium verticillioides* (≥ 98%, high performance liquid chromatography (HPLC)‐grade) and ATP (99% purity, HPLC‐grade, cat. no. A2385‐10G) were purchased from Merck (Dorset, UK). ATP disodium salt solutions of 100 mM ATP and 50 mM SA adjusted to pH 6.5 were prepared in water, while 1 mM FB1 was dissolved in 70% methanol. Control treatments contained the equivalent carrier solution lacking the chemical compound. Methods of cell culture and plant treatment, real‐time quantitative polymerase chain reaction (RT‐qPCR), and analysis of cell death are given in Supporting Information Methods S1.

### Construction of native and mutant RNS1 overexpressing plants

To create 2x*35S*:*RNS1* plasmid constructs, the full‐length coding sequence of At*RNS1* (At2g02990; 692 bp) was PCR‐amplified from mixed cDNA derived from siliques and flower buds using the primers 5′‐CACAAGCTTGAAAGATGAAGATTCTTCTAGC‐3′ and 5′‐CGACTGCAGAACTAAAAAGAAGGGAATTCGATCTCAGC‐3′. This was cloned in PCR‐Blunt II‐TOPO (Life Technologies, Paisley, UK). After digestion with *Hind*III and *Pst*I, the purified DNA fragment was sub‐cloned into vector pSAT6A‐EGFP‐N1 (Chung *et al*., 2005). Finally, the expression cassettes were sub‐cloned in the *PI‐Psp*I site of the cloning vectors pRCS2‐ocs‐nptII (destined for Col‐0 Arabidopsis) and pRCS2‐ocs‐bar (destined for *nahG* Arabidopsis) (Chung *et al*., 2005). The noncatalytically active mutant version of *RNS1* was generated using the QuickChange II XL Site‐Directed Mutagenesis Kit (Agilent Technologies, Stockport, UK), changing both residues 63 (primer 5′‐GCTGATTTTGGCATTTTCGGTCTTTGGCCTAAC‐3′) and 123 (primer 5′‐CACGAATGGGAGAAGTTTGGTACTTGCTCTGAATCG‐3′) from histidine to phenylalanine. Binary plasmids were transformed into *Agrobacterium tumefaciens* strain GV3101:pMP90 (Koncz & Schell, 1986), which was used for transformation of Arabidopsis plants (Clough & Bent, 1998).

### Proteomics and *RNS1* transcript analyses using cell suspension cultures

RNA was isolated from mock‐treated Arabidopsis cells and cells treated with 1 µM FB1 only, or a combination of 1 µM FB1 and 1 mM ATP (FB1+ATP), with the ATP having been added 40 h after the FB1. Cell culture samples were harvested at 40, 41, 42, 44, and 48 h after initiating the experiment by FB1 addition. Three biological replicates of each sample were generated. Real‐time quantitative polymerase chain reaction analysis for *RNS1* expression was performed across the time‐course using *ACTIN‐2* and *EIF4A* as constitutive reference controls, as described previously (Chivasa *et al*., 2006). For proteomic analysis, growth medium protein fractions were recovered from cell cultures at the 48‐h time point using a previously described method (Smith *et al*., 2015). Quantitative analysis was performed via two‐dimensional difference gel electrophoresis (2D‐DiGE) and the DeCyder software package (Alban *et al.*, 2003), using the two‐protein dye (Cy3 and Cy5) labelling system as described elsewhere (Chivasa *et al*., 2010). Four biological replicates were used for quantitative differential analysis via 2D‐DiGE. Proteins of interest were identified by MALDI‐TOF/MS on an Applied Biosystems (Foster City, CA, USA) 4800 mass spectrometer using a previously published protocol (Chivasa *et al*., 2013).

### Ribonuclease activity assays

RNase in‐gel activity assays were performed according to the method of Yen & Green (1991) with minor modifications. Leaf tissue was homogenised at a rate of 10 mg µl^−1^ extraction buffer (150 mM citric acid–Na_2_HPO_4_, pH 3.0, 0.1 mM phenylmethylsulfonyl fluoride (PMSF)) and the supernatant retained after centrifugation for 10 min at 10 000 ***g***. Protein concentration was assayed according to the method described by Bradford (1976). Samples with 20 µg total protein were mixed with a quarter volume of 5× sample‐loading buffer (5% (w/v) sodium dodecyl sulfate (SDS), 25% (w/v) glycerol, 0.125% (w/v) bromophenol blue). The samples were loaded into wells of a stacking gel with a resolving gel at the bottom. The resolving gel was: 14.61% (w/v) acrylamide, 0.39% (w/v) *N*,*N*′‐methylenebisacrylamide, 0.46 M Tris pH 9.0, 2.4 mg ml^−1^ *Torulopsis utilis* RNA (Sigma‐Aldrich), 0.2% (v/v) *N*,*N*,*N*′,*N*′‐tetramethyl‐ethylenediamine, and 0.05% (w/v) ammonium persulphate. The stacking gel had essentially the same composition, except for 5% (w/v) acrylamide, 0.125 M Tris (pH 6.8), and the absence of RNA. Proteins were electrophoresed until the dye‐front reached the bottom of the gels using the running buffer: 1.44% (w/v) glycine, 24.8 mM Tris, 0.1% (w/v) SDS. Gels were incubated for 2 × 10 min in wash buffer (25% (v/v) isopropanol, 10 mM Tris pH 6.0) followed by 2 × 10 min washes in 10 mM Tris pH 6.0 containing 2 µM ZnCl_2_. After incubation for 50 min at 55°C in 100 mM Tris pH 6.0, the gels were washed once for 10 min in 10 mM Tris pH 6.0 and incubated for 10 min in 0.2% (w/v) Toluidine blue, 10 mM Tris pH 6.0. The gels were de‐stained with several washes of 10 mM Tris pH 6.0 until clear bands where RNA had been digested were clearly visible against a blue background. Ribonuclease activity was imaged and the gels were completely de‐stained before Coomassie blue staining of the proteins and re‐imaging of the protein bands.

## Results

### Proteomic and transcriptional responses of *RNS1* after mycotoxin stress treatment

**Fig. 1:**
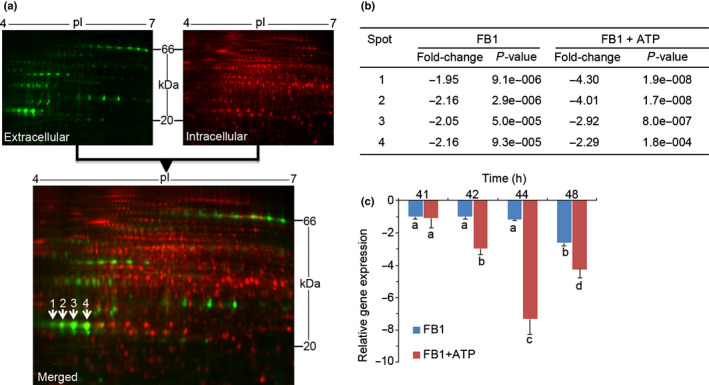
Effects of FB1 and ATP on Arabidopsis *RNS1* expression. (a) Two‐dimensional (2D) gel analysis of untreated Arabidopsis cell culture proteins secreted into the growth medium (Cy3‐labelled, green) and intracellular proteins (Cy5‐labelled, red). The overlay image reveals that RNS1 spots (numbered 1–4) and all other secreted proteins are enriched in the growth medium. (b) Two‐dimensional difference gel electrophoresis analysis of RNS1 protein spots. Fold‐change represents the ratio of average spot volume of fumonisin B1 (FB1)‐treated or FB1+ATP‐treated samples to mock‐treated controls. Averages and Student’s *t*‐test *P*‐values are based on four replicates. (c) Quantitative analysis of *RNS1* expression in samples treated with FB1 only or FB1+ATP. Gene expression is expressed relative to the FB1‐treated samples at 40 h. Data and error bars represent means ± SD (*n* = 3). Data were analysed by one‐way ANOVA and Tukey’s test at the 95% confidence level. Means that do not share a letter are significantly different (*P* ≤ 0.05). RNS1, RIBONUCLEASE 1.

In Arabidopsis cell suspension cultures, FB1‐triggered cell death can be blocked by exogenous ATP added up to *c*. 40 h after exposure to FB1, but ATP addition at/or later than *c*. 48 h fails to avert PCD (Chivasa *et al*., 2005). Thus, critical events irreversibly committing cells to death occur within the 40–48 h window, which provides a suitable experimental system for identifying ATP‐regulated stress proteins with key functions in cell death (Chivasa *et al*., 2011). We used this system in a proteomic screen designed to identify differentially expressed proteins via 2D‐DiGE. Cells were treated with FB1, and exogenous ATP was added 40 h later. Proteins secreted into the growth medium were harvested at 48 h for analysis by 2D‐DiGE. One of the differentially expressed proteins was identified by tandem mass spectrometry as RNS1 (Table 1). On 2D gels, RNS1 exists as four isoelectric point (pI) variants of *c*. 23 kDa (Fig. 1). However, at 230 amino acid residues in length, the full protein has a predicted molecular weight of 25.4 kDa and a pI of 4.88. The first 22 amino acids constitute a predicted signal peptide (von Heijne, 1990), targeting it to the secretory pathway. Signal peptide cleavage leaves a polypeptide of 207 amino acid residues of 22.97 kDa, which is consistent with the molecular mass observed on gels. RNS1 spots are abundant in the growth medium and are undetectable in total soluble protein extracts dominated by intracellular proteins (Fig. 1a).

**Table 1:** Details of Arabidopsis RNS1 protein identification by tandem mass spectrometry.

Spot^a^ number	Gene locus	Protein name	Matched^b^ peptides	Sequence coverage (%)	Sequenced peptides	Protein^c^ score
1	At2g02990	Ribonuclease 1	9	13	K.AGINPDGK.S	118
					K.TNLLGALTK.A	
					K.SYSLESIRDSIK.E	
2	At2g02990	Ribonuclease 1	5	9	K.SYSLESIR.D	110
					K.SYSLESIRDSIK.E	
3	At2g02990	Ribonuclease 1	9	21	K.TNLLGALTK.A	218
					K.SYSLESIR.D	
					K.SYSLESIRDSIK.E	
4	At2g02990	Ribonuclease 1	6	13	K.SYSLESIR.D	120
					K.SYSLESIRDSIK.E	

^a^ Spot numbers refer to RNS1 protein spots shown in Fig. 1.

^b^ Number of peptide masses matched to the theoretical digest of the RNS1 protein sequence.

^c^ Protein scores > 70 are significant (*P* < 0.05) as calculated in proteinpilot (https://sciex.com/products/software/proteinpilot‐software).

FB1 treatment downregulated all RNS1 spots, and exogenous ATP massively extended the reduction in protein abundance, with the magnitude of ATP‐induced change increasing inversely with decreasing protein pI (Fig. 1a,b). These results are consistent with *RNS1* gene expression profiling conducted over the 40–48 h window. Using the 40 h FB1 treatment as the baseline, *RNS1* transcript abundance did not change in the first 4 h of the 40–48 h window in cells treated with FB1 alone (Fig. 1c). A modest drop in transcript abundance was registered at the 48 h timepoint. By contrast, the addition of ATP to FB1‐treated cells swiftly suppressed *RNS1* transcript abundance nearly 8‐fold within the same 4 h period (Fig. 1c). A possible interpretation of these results is that RNS1 supports cell death signalling, which FB1‐treated cultures attempt and fail to curtail. However, the addition of exogenous ATP successfully downregulates *RNS1* and consequently rescues the cells.

**Fig. 2:**
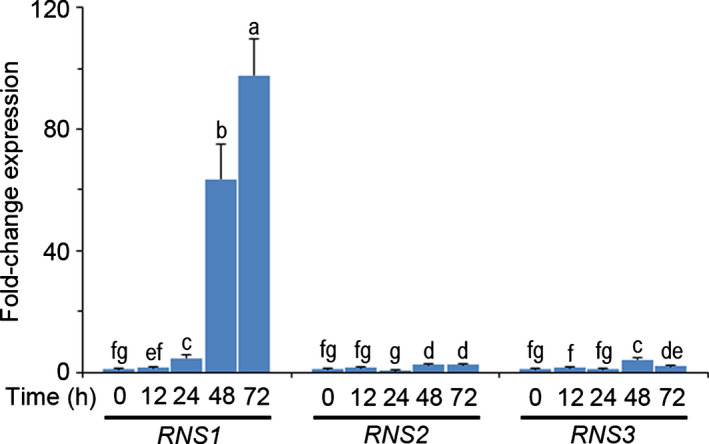
Arabidopsis *S*‐like RNase gene response to FB1 treatment. Quantitative analysis of *S*‐like RNase gene expression using RNA samples harvested at the indicated times from Arabidopsis cell cultures treated with 1 µM fumonisin B1 (FB1). Data and error bars represent means ± SD (*n* = 3). Data were analysed by one‐way ANOVA and Tukey’s test at the 95% confidence level. Means that do not share a letter are significantly different (*P* ≤ 0.05). *RNS1‐3, RIBONUCLEASE 1‐3*.

RNS1 belongs to an Arabidopsis five‐member gene family of *S* locus‐like ribonucleases (*S*‐like RNases): At2g02990 – *RNS1*, At2g39780 – *RNS2*, At1g26820 – *RNS3*, At1g14210 – *RNS4*, and At1g14220 – *RNS5*. In our experiments, we have been able to PCR‐amplify only *RNS1*, *RNS2*, and *RNS3* using cDNA from Arabidopsis cell cultures or leaf material. Root expression of *RNS4* has been reported previously (Megel *et al*., 2019), but there are currently no reports of *RNS5* expression to our knowledge. Therefore, to assess the *S*‐like RNase response to FB1 treatment, we restricted our analysis to the first three genes (*RNS1–RNS3*). While all genes responded to FB1 stress treatment, *RNS1* had the strongest response, reaching nearly 100‐fold induction (Fig. 2). These results show that *RNS1* is the most FB1‐responsive Arabidopsis *S*‐like RNase.

Considered together, the results shown in Figs 1 and 2 suggest complex regulation of RNS1 protein turnover during the stress response to FB1. When treated with FB1, *RNS1* expression initially increases and reaches a high peak around *c*. 40 h, where signalling events irreversibly committing cells to death are initiated. *RNS1* expression can be seen to be fall in the 40–48 h window (Fig. 1c) because the peak expression at 40 h serves as the baseline for revealing these critical events in this window. At the crucial 48 h timepoint, around which commitment to cell death is complete, RNS1 protein levels are lowered in FB1‐treated cells (Fig. 1b), implying that post‐translational control is recruited as a mechanism to regulate RNS1 protein turnover. This is despite the apparent residual transcript increase that lingers on, if the results are viewed from the 0 h timepoint as the baseline (Fig. 2). Thus, FB1‐treated cells unsuccessfully attempt to shut down RNS1 to avert death. Crucially, exogenous ATP rescues cells by rapidly curtailing *RNS1* gene expression and suppressing RNS1 protein abundance. Therefore, we proposed the hypothesis that RNS1 is a putative pro‐cell death protein that is targeted by ATP.

### *RNS1* is a stress response gene regulated by ATP and salicylic acid

**Fig. 3:**
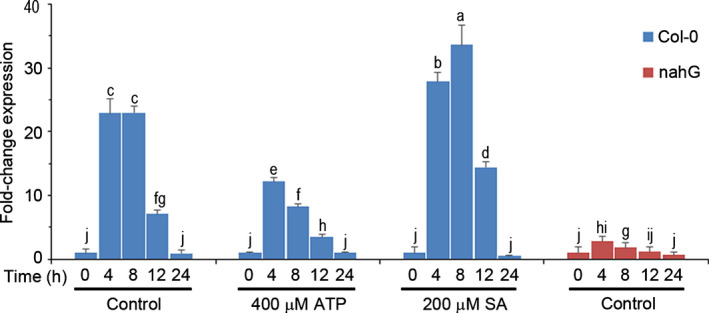
Effects of ATP and salicylic acid (SA) on wound‐induced *RNS1* expression. Leaves of Col‐0 plants were infiltrated with water (control), 400 µM ATP, or 200 µM SA, and samples for gene expression analysis were harvested at the indicated times. Leaves of *nahG*‐expressing plants were wounded by infiltration with water and samples for quantitative real‐time polymerase chain reaction (RT‐qPCR) analysis were harvested at the indicated timepoints. Data and error bars represent means ± SD (*n* = 3). Data were analysed by one‐way ANOVA and Tukey’s test at the 95% confidence level. Means that do not share a letter are significantly different (*P* ≤ 0.05). *RNS1, RIBONUCLEASE 1*.

Apart from responding to FB1 treatment (Fig. 2), *RNS1* expression is also activated by multiple stresses (GENEVESTIGATOR database, https://genevestigator.com/, accessed 5 January 2022), including wounding (LeBrasseur *et al*., 2002). ATP attenuates cell death activated by FB1 stress (Chivasa *et al*., 2005; Smith *et al*., 2021) and suppresses FB1‐induced *RNS1* gene expression in cell suspension cultures (Fig. 1c). We decided to investigate whether ATP affects stress‐induced *RNS1* expression beyond FB1 stress. Therefore, we assessed the impact of ATP on wound induction of *RNS1* expression using syringe‐infiltration of solutions into the leaf apoplast as a way to inflict damage. Comparison of wounding caused by water infiltration with damage inflicted by pressing a leaf between the ridged surfaces of a pair of forceps revealed very similar *RNS1* expression profiles over a 24‐h period (Fig. S1). The key difference between the two is that *RNS1* expression had returned to basal levels by 24 h in water‐infiltrated tissues, while it remained very high in forceps‐damaged leaves (Fig. S1). Providing contrast, *DORN1/P2K1* expression was consistently suppressed by both types of wounding damage across the entire time‐course, while *RNS1* expression was upregulated (Fig. S1). Consistent with a role for stress suppression, ATP infiltration significantly suppressed the *RNS1* activation seen in control solution‐infiltrated leaves across the 24‐h monitoring period (Fig. 3).

Having noted that water infiltration‐induced *RNS1* expression peaks in the first few h and declines to the basal level by 24 h, we evaluated the response to FB1 from 24 h onwards. FB1 infiltration activated *RNS1* expression, and co‐infiltration with ATP blocked this response (Fig. S2), confirming that the results obtained from cell cultures (Fig. 1c) can be reproduced in plants. More significantly, application of FB1 to hydroponic plants in the absence of wounding or any mechanical damage activated *RNS1* expression (Fig. S3). Taken together, these results show that both wound‐induced and FB1‐induced expression of *RNS1* are regulated by ATP.

The phytohormone SA regulates FB1 stress‐induced cell death in Arabidopsis. Exogenous SA enhances (Smith *et al*., 2015), while SA‐depleted transgenic plants expressing a bacterial salicylate hydroxylase gene (*nahG*) suppress, FB1‐induced death (Asai *et al*., 2000). We evaluated the impact of SA on wound‐induced *RNS1* expression by syringe‐infiltration of SA into wild‐type plants and a control solution into SA‐depleted *nahG*‐expressing plants. Exogenous SA significantly increased the peak of wound‐induced *RNS1* expression, while *nahG*‐driven depletion of SA completely blocked the *RNS1* wounding response (Fig. 3). Moreover, depletion of SA by salicylate hydroxylase blocked FB1‐induced *RNS1* expression in *nahG*‐plants (Fig. S4). Overall, these results show that both ATP and SA regulate the stress (wounding and mycotoxin) response of *RNS1*gene expression.

### Generation of transgenic Arabidopsis with altered RNS1 protein levels and enzymatic activity

**Fig. 4:**
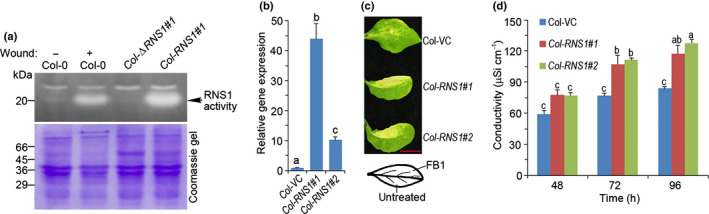
RNS1 activity in transgenic Arabidopsis plants alters the cell death response to FB1. (a) Upper panel: in‐gel ribonuclease activity of native protein extracts from control and wounded wild‐type Col‐0 plants alongside unwounded transgenic plants transformed with constructs expressing native (*Col‐RNS1#1*) or catalytically inactive mutant (*Col‐*Δ*RNS1#1*) RIBONUCLEASE 1 (RNS1) protein. Lower panel: Coomassie blue staining of the same gel showing equal protein loading across the samples. Numbers indicate positions of molecular weight markers. (b) Gene expression analysis of *RNS1* in vector control (VC) transgenic Col‐0 plants carrying an empty plasmid vector (Col‐VC) or two independent transgenic lines overexpressing *RNS1* (*Col‐RNS1#1* and *Col‐RNS1#2*). Gene expression is relative to the Col‐VC line. Data and error bars represent mean ± SD (*n* = 3). (c) Images of representative leaves infiltrated with 5 µM fumonisin B1 (FB1) and photographed 3 d later. Only the top half was FB1‐treated, as indicated in the leaf sketch. Bar, 13 mm. (d) Comparison of FB1‐induced cell death rates. Leaf discs were floated on 5 µM FB1, and conductivity of the FB1 solution was measured with time. Data and error bars represent mean ± SE (*n* = 4). Data in (b) and (d) were analysed by one‐way ANOVA and Tukey’s test at the 95% confidence level. Means that do not share a letter are significantly different (*P* ≤ 0.05).

To investigate a possible role for RNS1 in cell death, we generated two plasmid constructs expressing native RNS1 protein (*35S:RNS1*) or a catalytically inactive mutant (*35S:*Δ*RNS1*) driven by the constitutive 35S CaMV promoter. The enzymatically dead version of RNS1 was constructed by mutagenizing two highly conserved histidine residues in the catalytic core. Similar mutations in other T_2_‐RNase family members inactivate the enzyme (Deshpande & Shankar, 2002; Acquati *et al*., 2005; Thompson & Parker, 2009). To evaluate enzymatic activity in proteins encoded by these constructs, we transformed Arabidopsis (Col‐0 ecotype) and used an in‐gel ribonuclease assay of leaf protein extracts from representative plants. As positive controls to locate the RNS1 activity band, extracts from wounded wild‐type plants were included for visualisation of the endogenous wound‐induced activation of *RNS1*. At the acidic pH we used for the assay, we saw two ribonuclease bands and identified the lower band as RNS1 due to its stimulation with wounding and increased activity in transgenic *Col‐RNS1* lines (Fig. 4). The RNS1 activity band in these native gels runs at *c*. 20 kDa, which is lower than the 23 kDa seen in reducing gels (Fig. 1a). This indicates the presence of disulphide bridges within the secondary structure of the protein. The larger ribonuclease band running at a position higher than RNS1 did not change in response to wounding or overexpression of RNS1 and so served as an additional loading control. Even though *RNS1* expression in *Col‐*Δ*RNS1* plants was 4‐fold higher than in *Col‐RNS1* (Fig. S5), RNS1 enzymatic activity was detected only in plants overexpressing native RNS1, and no activity was detectable in extracts of transgenic plants transformed with the mutant RNS1 (Fig. 4a). This confirms that the mutant RNS1 protein is catalytically inactive. Therefore, these constructs were used to generate several transgenic Arabidopsis lines that were used in subsequent experiments. We did not detect any growth or developmental defects in any of the lines generated.

### RNS1 promotes FB1‐induced cell death

To evaluate the impact of RNS1 on FB1‐induced cell death, we used Col‐0 ecotype Arabidopsis transgenic lines carrying an empty plasmid vector (to serve as a control) and plants carrying the *35S:RNS1* construct expressing native RNS1. Plants from two independent representative lines (*Col‐RNS1#1* and *Col*‐*RNS1#2*) with 44‐fold and 10‐fold *RNS1* expression (Fig. 4b) relative to the empty vector control were infiltrated with 5 µM FB1 on one half of a leaf. Three days later, the treated half leaf of the vector control plants had developed the typical patchy cell death, while both lines with increased RNS1 activity had extensive cell death that had spread to cover nearly all of the treated leaf half (Fig. 4c). For quantitative cell death assays, we measured the conductivity of FB1 solutions on which leaf discs were floated. As cells die, electrolyte leakage leads to a measurable rise in conductivity of the underlying solution. The kinetic profiles of conductivity revealed that overexpression of *RNS1* significantly increased cell death in both transgenic lines (Fig. 4d).

**Fig. 5:**
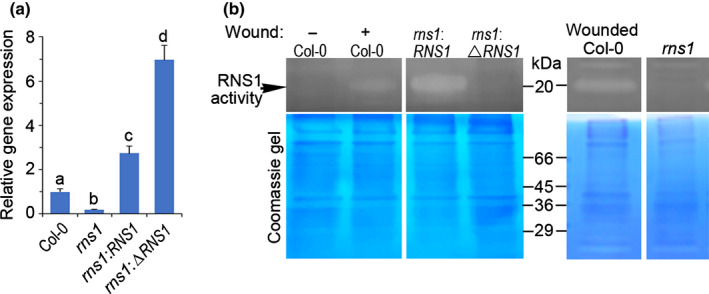
Characterisation of *rns1* gene knockout and complementation Arabidopsis lines. (a) Gene expression analysis of *RNS1* in *rn1* mutant plants and *rns1* complementation lines expressing the native (*35S:RNS1*) or loss‐of‐function (*35S:*Δ*RNS1*) plasmid constructs. Data and error bars represent mean ± SD (*n* = 3). Data were analysed by one‐way ANOVA and Tukey’s test at 95% confidence interval. Means that do not share a letter are significantly different (*P* ≤ 0.05). (b) Upper panels: RNS1 ribonuclease activity in extracts of plants from the indicated lines relative to the control and forceps‐wounded Col‐0 plants. Lower panels: Coomassie blue‐stained gels showing protein loading across the samples. Positions of molecular weight markers and the RNS1 activity are indicated. RNS1, RIBONUCLEASE 1.

A T‐DNA insertion mutant line (SALK_087165.56.00.X) from the SALK collection (Alonso *et al*., 2003) was used to further explore the role of RNS1 in cell death. The T‐DNA in this *rns1* mutant is in the 5′‐untranslated region of the *RNS1* gene and blocks stress induction of the gene, causing a 5‐fold difference in transcript abundance in comparison to the wild‐type after wounding with a pair of forceps (Fig. 5a). Suppression of gene induction is reflected by a lack of RNS1 enzyme activity in protein extracts from *rns1* plants (Fig. 5b). We complemented *rns1* plants with a plasmid carrying the *35S:RNS1* construct to express the native RNS1 protein or the *35S*:Δ*RNS1* construct for the expression of an enzymatically dead version of the protein. The *rns1:RNS1* and *rns1:* Δ*RNS1* lines increased *RNS1* expression nearly 3‐fold and 7‐fold, respectively, in the mutant background (Fig. 5a). As expected, complementation with native RNS1 restored RNS1 enzyme activity in protein extracts, while no activity was seen in plants complemented with the dead version (Fig. 5b).

**Fig. 6:**
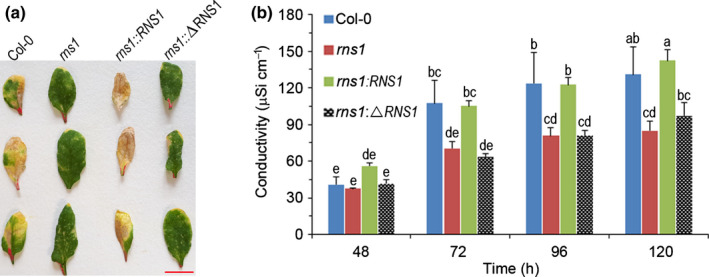
Cell death in response to FB1 of *rns1* gene knockout and complementation Arabidopsis lines. (a) Representative leaves infiltrated with 5 µM fumonisin B1 (FB1) and detached from plants 7 d later for photographing. Bar, 26 mm (b) Leaf discs were floated on 5 µM FB1 and conductivity of the FB1 solution was measured over time. Data and error bars represent mean ± SE (*n* = 4). Data were analysed by one‐way ANOVA and Tukey’s test at the 95% confidence level. Means that do not share a letter are significantly different (*P* ≤ 0.05). RNS1, RIBONUCLEASE 1.

Leaves from wild‐type (Col‐0), *rns1* mutant, and complementation plants were treated with 5 µM FB1 by syringe‐infiltration of the entire leaf. Representative leaves were detached and photographed 7 d later (Fig. 6a). The first patchy cell death symptoms appeared in Col‐0 leaves 3 d later and progressively spread with time. The T‐DNA mutants were essentially symptom‐free at 3 d, and the leaves remained alive at the end of the 7 d. Leaves complemented with native RNS1 had more severe symptoms than the wild‐type and died much earlier, a result similar to what we observed in Fig. 4(c). Crucially, the dead version of RNS1 lacking any enzyme activity failed to restore the cell death response in complemented *rns1* mutants (Fig. 6a). The quantitative leaf disc assay confirmed that loss of RNS1 function suppresses cell death, and complementation with native RNS1, but not the catalytically inactive version, restores the cell death response (Fig. 6b). However, there is a subtle difference between the FB1 responses of *rns1:RNS1* plants observed in the leaf infiltration assay and the conductivity assay. In infiltrated leaves, cell death was consistently more extensive in *rns1:RNS1* plants than in wild‐type plants, while in the conductivity assay the cell death level was the same (Fig. 6b). This reflects minor differences between the *in planta* and *in vitro* experimental systems utilised here. Overall, these results suggest that RNS1 enzymatic activity is essential for FB1‐induced cell death.

We also obtained *asRNS1* transgenic plants expressing an antisense *RNS1* construct (Bariola *et al*., 1999). Although we did not see significant differences between the responses of wild‐type Col‐0 plants and *asRNS1* plants to 5 µM FB1, we found differences when the amount of FB1 was reduced to below 1 µM. At the low FB1 concentrations, we used the lactophenol‐blue staining method to detect cell death at the single‐cell level via light microscopy. We detected cell death lesions on leaves from Col‐0 plants from a concentration as low as 0.2 µM, with the lesions increasing in density when FB1 concentration was doubled to 0.4 µM and then to 0.8 µM (Fig. S6). By contrast, *asRNS1* plants were immune to FB1 at the low concentrations and only capitulated at 0.8 µM FB1 (Fig. S6). Considered together, these results show that high concentrations of RNS1 enhance FB1‐induced cell death, while reduced enzyme activity suppresses death.

### *RNS1* overexpression alters the response of *nahG*‐expressing plants to FB1

**Fig. 7:**
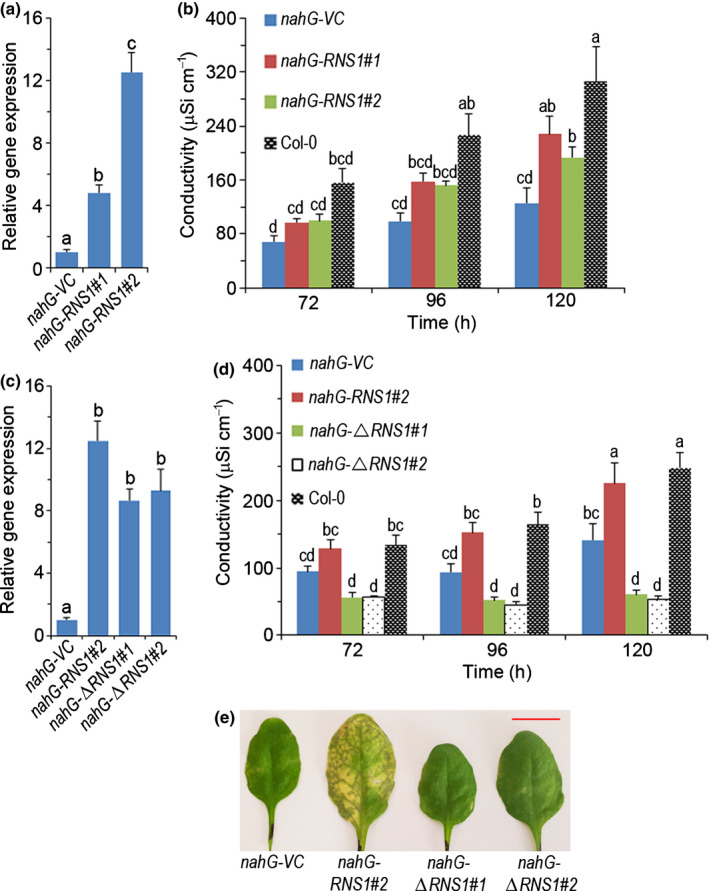
Response to FB1 of Arabidopsis wild‐type and *nahG*‐plants with altered RNS1 levels. (a) *RNS1* expression in *nahG*‐plants transformed with an empty vector control (VC) plasmid and two independent lines of *nahG*‐plants transformed with a plasmid expressing 35S CaMV promoter‐driven *RNS1*. (b) Leaf discs of indicated genotypes were floated on 5 µM fumonisin B1 (FB1) and conductivity of the solution measured at the indicated times. (c) *RNS1* expression *in nahG*‐plants transformed with an empty vector control plasmid or plasmids carrying a construct for either native RNS1 (*35S:RNS1*) or a catalytically inactive RNS1 mutant (*35S:*Δ*RNS1*). (d) Leaf discs from the indicated genotypes were floated on 5 µM FB1, and the conductivity of the solution was measured at the indicated times. Data and error bars represent mean ± SD (*n* = 3) for gene expression or mean ± SD (*n* = 5) for leaf disc assays. Data in (a–d) were analysed by one‐way ANOVA and Tukey’s test at the 95% confidence level. Means that do not share a letter are significantly different (*P* ≤ 0.05). (e) Appearance of representative leaf discs 7 d after infiltration with 5 µM FB1. Bar, 1.4 cm. RNS1, RIBONUCLEASE 1.

Salicylic acid‐deficient *nahG*‐expressing plants (Lawton *et al*., 1995) suppress stress induction of RNS1 (Fig. 3) and are resistant to FB1 stress‐induced death (Asai *et al*., 2000). We wanted to investigate whether suppression of *RNS1* induction could account for the resistance of *nahG*‐plants to FB1. Therefore, we transformed *nahG*‐plants with an empty plasmid vector or a vector carrying the *35S:RNS1* construct to overexpress native RNS1 protein. To illustrate the impact of RNS1 on FB1 responses in the *nahG*‐expressing plants, we selected two lines (*nahG‐RNS1#1* and *nahG‐RNS1#2*) with low (5‐fold) and high (12‐fold) basal *RNS1* expression levels (Fig. 7a). Quantitative analysis of cell death using the electrolyte leakage assay showed a significant increase in cell death when compared to the *nahG*‐expressing plants transformed with the empty vector (Fig. 7b). However, the susceptibility of the *nahG‐RNS1#1* and *nahG‐RNS1#2* Arabidopsis lines to FB1 remained significantly lower than Col‐0 (Fig. 7b), indicating that other SA‐dependent factors work in conjunction with RNS1 in establishment of PCD.

We generated further *nahG*‐expressing lines transformed with the *35S:*Δ*RNS1* construct expressing the catalytically dead enzyme. We selected two independent lines (*nahG‐*Δ*RNS1#1 and nahG‐*Δ*RNS1#2)*, both of which exhibited a *c*. 9‐fold increase in *RNS1* transcript accumulation (Fig. 7c). In a leaf disc conductivity assay experiment, the positive control line (*nahG‐RNS1#2*) overexpressing native RNS1 protein showed significantly increased cell death in comparison to the empty vector control line (Fig. 7d). We note that in comparison with Col‐0, the cell death level was statistically not different (Fig. 7d), unlike in the experiment shown in Fig. 7(b). We later realised that this discrepancy is plant age‐dependent, with FB1 activating more rapid and extensive cell death in younger (3–4 wk) Col‐0 plants than older (5–6 wk) plants. Use of a broad age range ensures that the results obtained are not plant age‐specific, but are applicable across growth stages. Surprisingly, both lines overexpressing the catalytically dead RNS1 protein significantly suppressed conductivity below the control line transformed with an empty vector (Fig. 7d). This is likely due to competitive exclusion of native RNS1 protein, by the overexpressed catalytically inactive mutant, from substrates. The appearance of representative leaves 7 d after infiltration with 5 µM FB1 (Figs 7e, S7) shows a difference in the death symptoms between the Col‐0 ecotype and *nahG*‐plants. Overall, these results further strengthen the evidence for a role of RNS1 in promoting FB1‐induced cell death via RNA cleavage.

### Link between *RNS1* and *PLCL1* expression

We previously identified PLCL1 (At1g13680) as a key protein required for FB1‐induced cell death. T‐DNA knockout mutants unable to express *PLCL1* are immune to the mycotoxin and suppress FB1‐induced cell death (Smith *et al*., 2021). To better understand the relationship between *RNS1* and *PLCL1*, we measured *RNS1* expression in the T‐DNA knockout mutants *plcl1‐1* and *plcl1‐2* lacking the PLCL1 protein. *RNS1* expression was massively suppressed in the mutants (Fig. S8), suggesting that the FB1 resistance seen in these mutants is mediated via *RNS1* suppression. *RNS1* overexpression lines showed a very modest increase in *PLCL1* expression (Fig. S8). These results indicate that RNS1 is likely downstream of PLCL1 in cell death signalling.

## Discussion

The cell death‐regulatory role of eATP in plant stress was investigated using proteomics as a primary screen. The screen identified RNS1 as a key target of eATP, and subsequent experiments revealed a previously unknown cell death function of RNS1. Three key observations provided crucial evidence revealing the role of RNS1 in stress‐induced death. First, FB1 activated rapid cell death that spread more extensively in transgenic plants with increased RNS1 protein, while *rns1* knockout plants and antisense *RNS1* plants had reduced sensitivity to FB1. The second observation was the abolition of FB1‐resistance in *nahG*‐expressing plants transformed with a construct expressing *35S:RNS1*. Third, a catalytically inactive mutant of RNS1 lacking ribonuclease activity neither complemented *rns1* gene knockout mutants nor restored the cell death response to FB1 in *nahG*‐expressing plants. Because in certain rare circumstances point‐mutations may result in different protein localisation (Xiao *et al*., 2017), whether the RNS1 catalytic mutant localises to the same subcellular compartment will require verification to further understand these results.

RNS1 belongs to the ubiquitous T_2_‐ribonuclease (enzyme commission number (EC) 3.1.27.1) family, which has two subfamilies in plants: the *S*‐locus ribonuclease (*S*‐RNase) subfamily with a role in the prevention of self‐pollination, and the *S*‐like RNase subfamily found in all plant species (Igic & Kohn, 2001; MacIntosh *et al*., 2010). RNS1 is one of five genes in the Arabidopsis *S*‐like RNase subfamily, whose physiological functions have been elusive until now. *RNS1* expression is activated by diverse biotic and abiotic stresses (GENEVESTIGATOR database, https://genevestigator.com/). The breadth of stress stimuli activating expression of *RNS1* may indicate the fundamental importance of this gene across multiple plant stress responses.

Our results show that RNS1 ribonuclease activity is essential for its function in cell death. Activation of PCD and cytotoxicity by ribonuclease activity has been reported before in other systems. For example, the cytotoxic lectin α‐sarcin from *Aspergillus giganteus* is an RNase which targets 28S ribosomal RNA (Ackerman *et al*., 1988). Colacin Df13 and colicin E3 are bacterial cytotoxic RNases that target 16S ribosomal RNA (Konisky, 1982). Onconase, the cytotoxic *Rana pipiens* ribonuclease, activates cell death by degradation of 28S and 18S ribosomal RNA (Wu *et al*., 1993). In plants, the rice probenazole‐induced protein1 has ribonuclease activity and activates PCD when expressed in Arabidopsis or when the recombinant protein is added to tobacco BY‐2 cell suspension cultures or infiltrated into the leaf apoplast of tobacco plants (Kim *et al*., 2011).

Plant *S*‐RNases terminate self‐pollen by RNA degradation (McClure *et al*., 1990), which disrupts protein translation (Gray *et al*., 1991) and results in death. Loss of ribonuclease activity disables the ability of *S*‐RNases to terminate self‐pollen (Huang *et al*., 1994). However, some reports indicate that for termination of self‐pollen, pollen tube RNA degradation occurs concurrently with additional signalling processes that promote PCD. *S*‐RNases alter reactive oxygen species and Ca^2+^ signalling to activate actin depolymerisation (Liu *et al*., 2007), paving the way to mitochondrial cytochrome *c* release into the cytosol and DNA fragmentation (Wang *et al*., 2009). Indeed, the application of drugs that can depolymerise actin is sufficient to induce PCD in pollen (Thomas *et al*., 2006) and some animal cells (Morley *et al*., 2003).

While there is a possibility that RNS1 triggers cell death via wholesale cellular RNA degradation, alternative mechanisms involving specific regulation of downstream genes may exist. For example, recent work has demonstrated that RNS1 cleaves tRNA *in planta* to produce a diverse population of tRNA‐derived fragments (tRFs) (Megel *et al*., 2019; Gu *et al*., 2022). In animals, tRFs regulate gene expression in diverse processes, including stress response, tumour suppression, and control of protein synthesis and apoptosis (reviewed by Kumar *et al*., 2016). Gu *et al*. (2022) demonstrated that a specific RNS1‐generated tRF associates with AGO1 to direct sequence‐specific degradation of *Cytochrome P450 71A13* transcripts for default suppression of anti‐fungal defence responses. These results imply that RNS1 is internalised into the cytoplasm and has functional roles in regulating gene expression. This aligns with our results showing that upregulation of *RNS1* expression is accompanied by increased expression of *PLCL1* (Fig. S8), a protein that enhances FB1‐induced cell death (Smith *et al*., 2021). Moreover, FB1 is known to change cell membrane fluidity and increase extracellular protein uptake in treated cells (Ferrante *et al*., 2002).

Our study shows that ribonuclease cell death function has been conserved between self‐incompatibility *S*‐RNases found in three plant families (Solanaceae, Rosaceae, and Scrophulariaceae) and *S*‐like RNases, which are prevalent in all plants, including self‐fertile plants. More importantly, we have shown that ATP abolishes FB1‐induced cell death by targeting the *RNS1* gene for suppression. Furthermore, SA regulates FB1‐induced cell death by targeting *RNS1* expression. Therefore, RNS1 is a convergence point for both salicylic acid and ATP signalling in the control of stress‐induced cell death. The discovery that RNA cleavage is an ATP‐regulated step in stress‐induced cell death presents RNS1 orthologues in crops as promising targets for crop improvement. Finally, our findings demonstrate the evolutionary conscription of plant T2‐RNases as cytotoxins for two distinct purposes – regulation of sexual reproduction (*S*‐RNases) and stress‐adaptive responses (*S*‐like RNases).

## Author contributions

SC designed and supervised the research and wrote the manuscript; JTMK generated all transgenic lines; HLG, DFAT, JMUH and AOA performed experiments.

## Supporting information


**Fig. S1** Effects of different methods of mechanical damage on wound gene expression.
**Fig. S2** Exogenous ATP suppresses FB1‐induced *RNS1* expression *in planta*.
**Fig. S3** FB1 activates *RNS1* expression in the absence of mechanical damage.
**Fig. S4** Enzymatic depletion of salicylic acid (SA) blocks the activation of *RNS1* expression by FB1.
**Fig. S5** Analysis of *RNS1* expression levels in transgenic plants.
**Fig. S6** Transgenic antisense RNS1 plants have reduced sensitivity to FB1‐induced cell death.
**Fig. S7** Appearance of leaves from transgenic plants 1 wk after FB1 treatment.
**Fig. S8** Interdependence of *RNS1* and *PLCL1* expression.
**Methods S1** Plant growth conditions and treatments.
Please note: Wiley Blackwell are not responsible for the content or functionality of any Supporting Information supplied by the authors. Any queries (other than missing material) should be directed to the *New Phytologist* Central Office.

## Data Availability

Additional data supporting this research are available on request from the corresponding author.
