# Supplementary material for: Extracellular ATP targets Arabidopsis RIBONUCLEASE 1 to suppress mycotoxin stress‐induced cell death
Source: New Phytol. 2022 May 31;235(4):1531–42. doi: 10.1111/nph.18211 (PMC9545236; doi:10.1111/nph.18211)
Supplement: Supplementary file 1 — Fig. S1 Effects of different methods of mechanical damage on wound gene expression. Fig. S2 Exogenous ATP suppresses FB1‐induced RNS1 expression in planta. Fig. S3 FB1 activates RNS1 expression in the absence of mechanical damage. Fig. S4 Enzymatic depletion of salicylic acid (SA) blocks the activation of RNS1 expression by FB1. Fig. S5 Analysis of RNS1 expression levels in transgenic plants. Fig. S6 Transgenic antisense RNS1 plants have reduced sensitivity to FB1‐induced cell death. Fig. S7 Appearance of leaves from transgenic plants 1 wk after FB1 treatment. Fig. S8 Interdependence of RNS1 and PLCL1 expression. Methods S1 Plant growth conditions and treatments. Please note: Wiley Blackwell are not responsible for the content or functionality of any Supporting Information supplied by the authors. Any queries (other than missing material) should be directed to the New Phytologist Central Office. [file NPH-235-1531-s001.pdf]

New Phytologist

New Phytologist **Supporting Information**

Article title: Extracellular ATP targets Arabidopsis RIBONUCLEASE 1 to suppress mycotoxin stress-induced cell death
Authors: Heather L. Goodman, Johan T. M. Kroon, Daniel F. A. Tomé, John M. U. Hamilton, Ali O. Alqarni, and Stephen Chivasa
Article acceptance date: 29 April 2022

The following Supporting Information is available for this article:

Fig. S1 Effects of different methods of mechanical damage on wound gene expression.

Fig. S2 Exogenous ATP suppresses FB1-induced *RNS1* expression *in planta.*

Fig. S3 FB1 activates *RNS1* expression in the absence of mechanical damage.

Fig. S4 Enzymatic depletion of salicylic acid blocks activation of *RNS1* expression by FB1.

Fig. S5 Analysis of *RNS1* expression levels in transgenic Arabidopsis plants.

Fig. S6 Transgenic antisense RNS1 plants have reduced sensitivity to FB1-induced cell death.

Fig. S7 Appearance of leaves from transgenic Arabidopsis plants a week after FB1 treatment.

Fig. S8 Interdependence of *RNS1* and *PLCL1* expression.

Methods S1 Plant growth conditions, treatments, and analyses

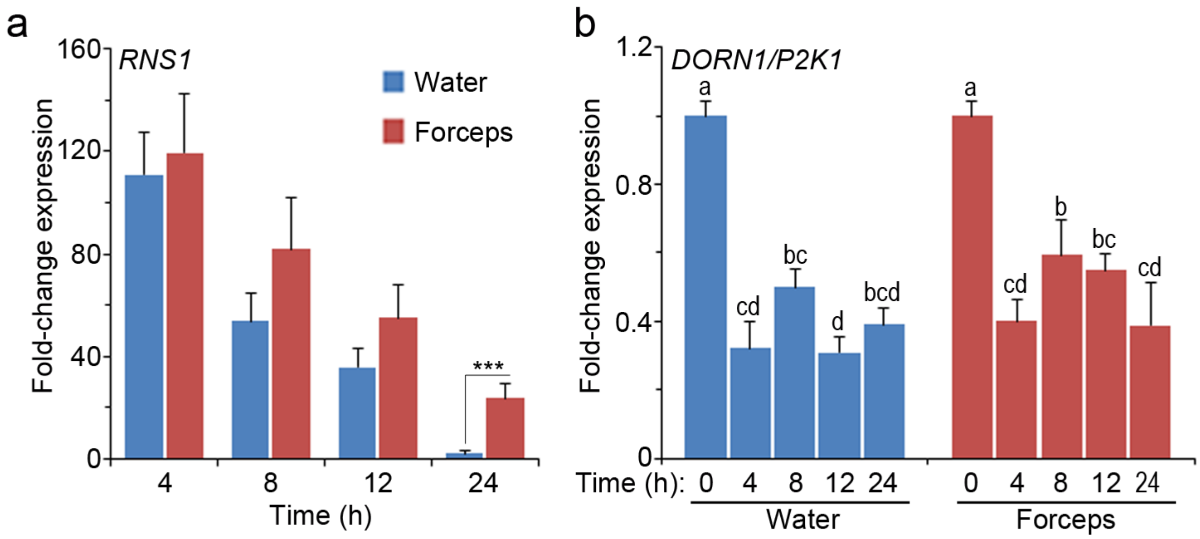

Fig. S1 Effects of different methods of mechanical damage on Arabidopsis wound gene expression. (**a**) Time-course of *RNS1* expression in Col-0 plants mechanically wounded by water infiltration into the leaf apoplast or leaf damage by pressing between the ridged surfaces of a pair of forceps. Asterisks indicate statistically significant ($P < 0.001$) differences between the mean for water-treated samples and forceps-damaged samples at the specified timepoint. (**b**) *DORN1/P2K1* expression in the same tissues used in **a**. Data and error bars represent mean ± SD ($n = 3$). Data were analysed by one-way ANOVA and Tukey's test at 95% confidence interval. Means that do not share a letter are significantly different ($P \leq 0.05$). *RNS1, RIBONUCLEASE 1; DORN1/P2K1, DOES NOT RESPOND TO NUCLEOTIDES 1/PURINORECEPTOR KINASE 1*.

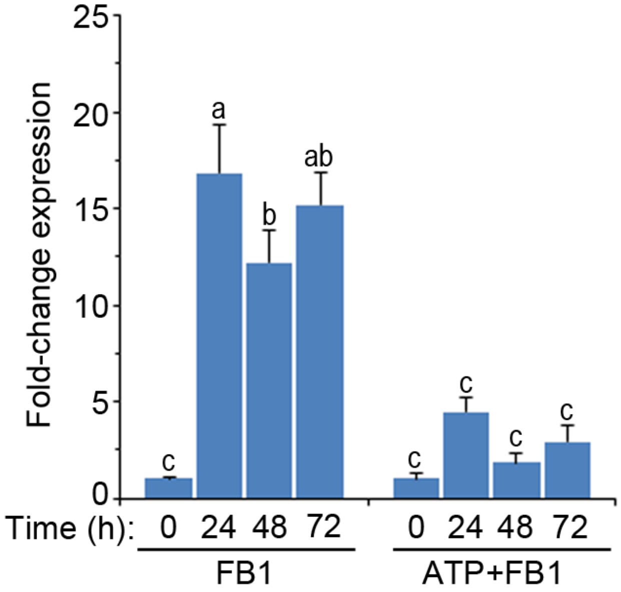

Fig. S2 Exogenous ATP suppresses FB1-induced *RNS1* expression *in planta*. Leaves of soil-grown plants were infiltrated with 5 µM fumonisin B1 (FB1) or 400 µM ATP mixed with FB1. Samples were harvested at the indicated timepoints for RNA extraction and quantitative gene expression analysis. Data and error bars represent mean ± SD (n = 3). Data were analysed by one-way ANOVA and Tukey's test at 95% confidence interval. Means that do not share a letter are significantly different ($P \leq 0.05$). *RNS1, RIBONUCLEASE 1*.

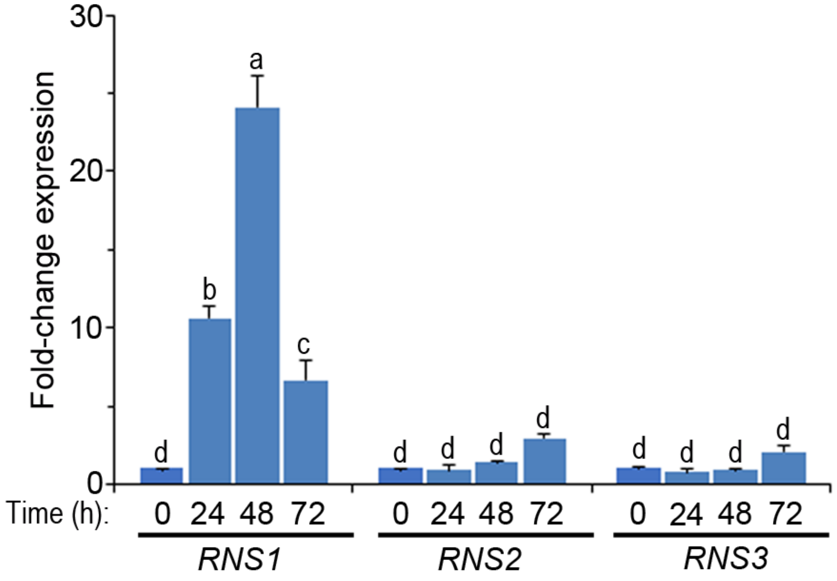

Fig. S3 FB1 activates *RNS1* expression in the absence of mechanical damage. Plants grown on solid agar were transferred to liquid medium and treated with 5 µM fumonisin B1 in the absence of mechanical injury for the indicated length of time. Expression of *RNS1-3* were analysed by real-time RT-PCR analysis. Data and error bars represent mean ± SD (n = 3). Data were analysed by one-way ANOVA and Tukey's test at 95% confidence interval. Means that do not share a letter are significantly different ($P \leq 0.05$). *RNS1-3, RIBONUCLEASE 1-3.*

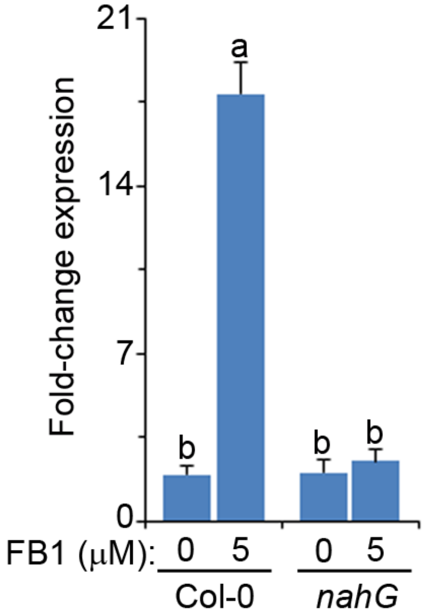

Fig. S4 Enzymatic depletion of salicylic acid blocks activation of *RNS1* expression by FB1. Arabidopsis wildtype Col-0 and transgenic *nahG*-plants were infiltrated with 5 μM fumonisin B1 or carrier solution and samples for RNA extraction taken 24 h after. *RNS1* expression was analysed by quantitative real-time RT-PCR analysis. Data and error bars represent mean ± SD (*n* = 3). Data were analysed by one-way ANOVA and Tukey's test at 95% confidence interval. Means that do not share a letter are significantly different ($P \leq 0.05$). *RNS1, RIBONUCLEASE 1*.

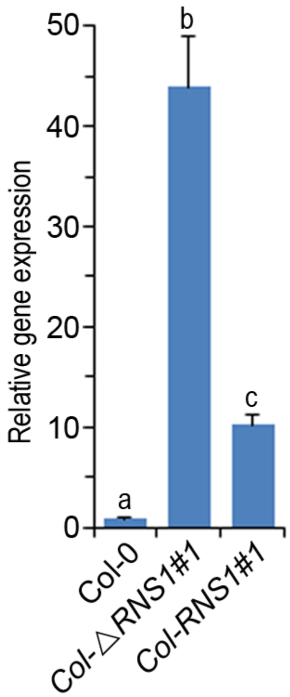

Fig. S5 Analysis of *RNS1* expression levels in transgenic Arabidopsis plants. RT-PCR analysis showing *RNS1* transcript abundance in transgenic plants, transformed with plasmid constructs for expression of native (*35S:RNS1*) or catalytically inactive (*35S:△RNS1*) RNS1, relative to wildtype Col-0 plants. Data and error bars represent means ± SD (n = 3). Data were analysed by one-way ANOVA and Tukey's test at 95% confidence interval. Means that do not share a letter are significantly different ($P \leq 0.05$). RNS1, RIBONUCLEASE 1.

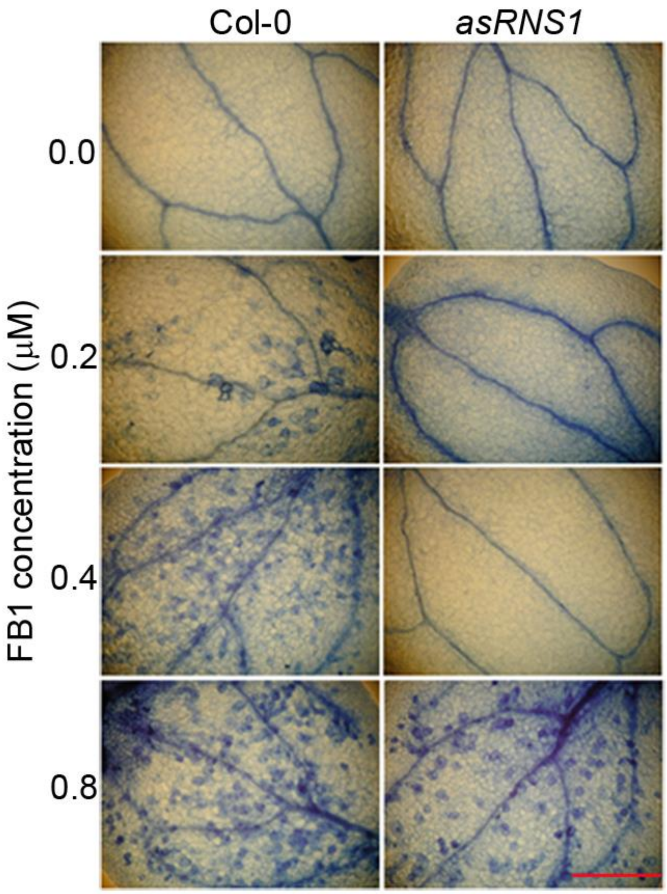

Fig. S6 Transgenic antisense *RNS1* plants have reduced sensitivity to FB1-induced cell death. Hydroponic wildtype (Col-0) and transgenic antisense-*RNS1* (*asRNS1*) Arabidopsis plants grown for 7 days were treated with FB1 at the indicated concentration for 5 days and stained with Evans blue. Photographed leaves are representative of leaves from a minimum of 20 replicate plants. Scale bar represents 4 mm. *RNS1, RIBONUCLEASE 1*.

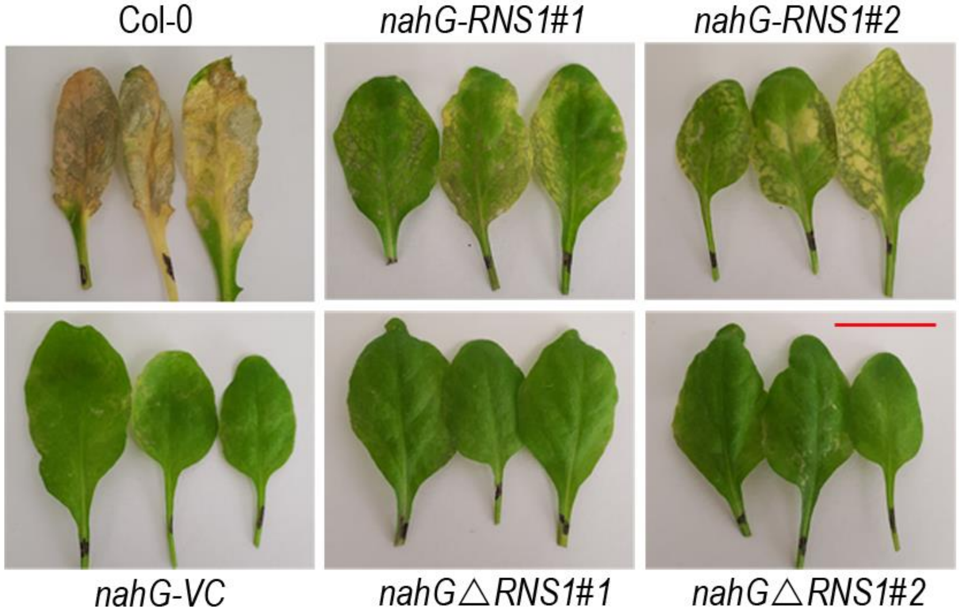

Fig. S7 Appearance of leaves from transgenic Arabidopsis plants a week after FB1 treatment. Leaves of soil-grown plants of the indicated genotypes were infiltrated with 5 µM FB1. After 7 days, representative leaves were photographed. Leaves are representative of 27 similar leaves from 9-replicate plants of each genotype. Scale bar represents 14 mm. *RNS1, RIBONUCLEASE 1*.

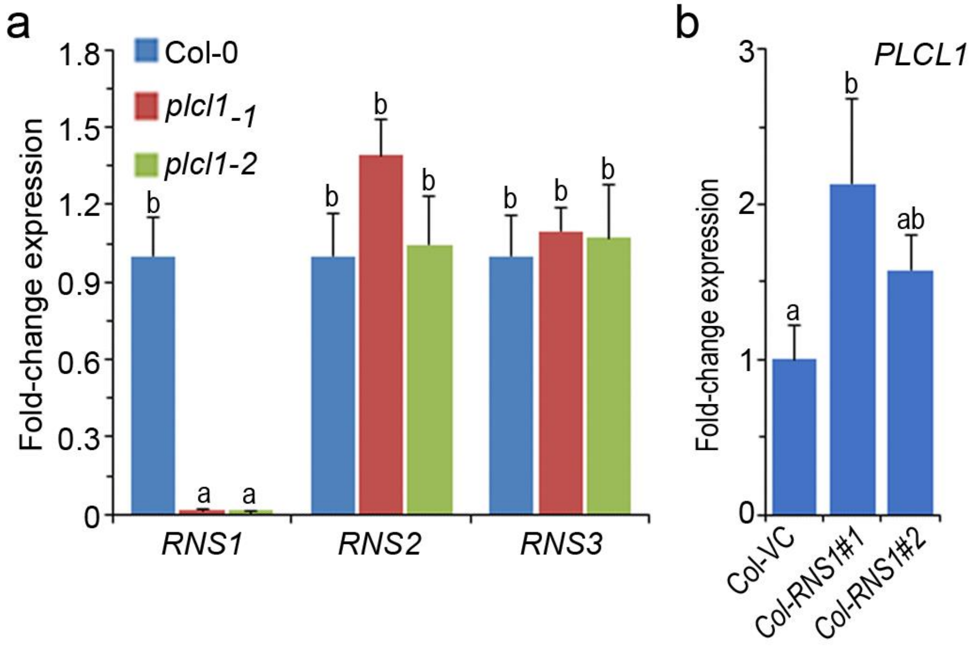

Fig. S8 Interdependence of *RNS1* and *PLCL1* expression. (a) *RNS1* expression is suppressed in T-DNA knockout plants lacking functional PLCL1. Leaf samples for RNA extraction and quantitative gene expression analysis were obtained from untreated soil-grown wildtype (Col-0) plants and T-DNA knockout mutants *plcl1-1* and *plcl1-2*. (b) *PLCL1* expression is upregulated in transgenic plants with raised levels of RNS1. Leaf samples for RNA extraction and quantitative gene expression analysis were obtained from untreated soil-grown wildtype (Col-0) and transgenic plants overexpressing native RNS1. Data and error bars represent means ± SD (*n* = 3). Data were analysed by one-way ANOVA and Tukey's test at 95% confidence interval. Means that do not share a letter are significantly different ($P \leq 0.05$). *RNS1-3, RIBONUCLEASE 1-3; PLCL1, PHOSPHOLIPASE C-LIKE 1.*

New Phytologist

Methods S1 Plant growth conditions, treatments, and analyses

Light regime

Plants used in experiments with results reported in Fig. 1-7 (main manuscript) were grown in a 16 h photoperiod at ~70 µmoles.m^{-2}.s^{-1}, while all plants used in experiments within Fig. S1-S8 were grown at a higher light intensity of ~120 µmoles.m^{-2}.s^{-1}.

Treatments

Soil-grown plants were treated by syringe-infiltration of FB1, SA or ATP solutions at concentrations indicated in the results and controls were mock-treated with the appropriate carrier solution. Infiltration was from the lower leaf surface, enabling the solutions to enter the apoplast through stomata. For wounding experiments, leaves were syringe-infiltrated with water or alternatively damaged by pressing between the ridged surfaces of a pair of forceps. For all treatments, 3 leaves per plant were treated and a single leaf from 3 independent plants harvested and pooled to make a single biological replicate per timepoint. Three such biological replicates were generated per timepoint. Leaves from untreated plants were similarly harvested to make generate biological replicates for the 0 h timepoint. For treatment of cell suspension cultures, solutions were filter-sterilized through a 0.2 µM nitrocellulose filter and added directly to the growth medium to the desired final concentration indicated in the results. For experiments to evaluate FB1 induction of *RNS1-RNS3* gene expression, 3 biological replicate cell cultures were generated per timepoint for RNA extraction.

Tissue culture plants were used to investigate if FB1 activates *RNS1* expression in the absence of tissue damage. Surface-sterilised seeds were germinated on Murashige and Skoog basal medium with solid agar (Chivasa *et al*., 2005) and grown for 10-14 days. Between 25-30 plants were transferred from agar plates into 10 mL of the same medium without agar in sterile flasks. The cultures were treated with filter-sterilised FB1 at a final concentration of 5 µM and the flask incubated without orbital shaking to avoid mechanical stimulation. Triplicate flasks were harvested at 24, 48, and 72 h after the start of treatment for RNA extraction Mock-treated samples served as controls.

Cell death assays

One cm-diameter leaf discs cored from soil-grown plants were floated on 5 µM FB1 in 4-replicate petri-dishes. A single dish contained 10 discs, each coming from one of 10 different plants. After a 48 h dark incubation, the dishes were returned to a 16 h photoperiod and conductivity of the solution measured immediately and every 24 h thereafter. The 48 h dark incubation enables the tissues to take up FB1 without activating cell death, which requires light. In other experiments, leaves attached to plants were infiltrated either on one half or both halves with 5 µM FB1. Three leaves per plant of 8-9 replicate plants were thus treated. Representative treated leaves were detached for photographing 3 days or 7 days later.

For qualitative cell death measurements using hydroponic plants, surface-sterilised seeds were germinated and grown in Murashige and Skoog basal liquid medium in sterile conical flasks on an orbital shaker, as described previously described (Chivasa *et al*., 2005). At 5 days old, the plants were treated with a final concentration of 0, 0.2, 0.4, or 0.8 µM FB1. Four-replicate flasks with 5 mL growth medium containing 5-8 plants each were generated at every FB1 concentration. After 5 days of treatment, the plants were stained with lactophenol-Evans blue (10 mL of lactic acid, 10 mL of glycerol, 10 g of phenol, 10 mg Evans blue, 10 mL deionised water, 80 ml ethanol) and de-stained with 2.5 mg/mL chloral hydrate. Representative leaves were photographed under a light microscope.

Quantitative RT-PCR analyses

RNA extraction and reverse transcription were performed as previously described (Chivasa *et al*., 2006). Three biological replicate samples, each consisting of leaves pooled from 3 independent plants, were generated for RNA extraction and used in real-time RT-PCR analyses. For cell suspension cultures, 3 independent cell cultures were setup to generate the 3 biological replicates. *ACTIN-2* and *EIF4A* were used as constitutive reference controls. The following primers were used: for *RNS1* (At2g02990), 5’-ATCCCGGCTTTGGTTAGAGC-3’ and 5’-CGTTTTGGGAGCACGAATGG-3’; for *RNS2* (At2g39780), 5’-TTTTAAGCCCAGGGACTGTG-3’ and 5’-

AGCTCCCATCTTTTCCGATT-3’; for *RNS3* (At1g26820), 5’-TGAGTCCGAGCTTGACCAAC-3’ and 5’-TCATCCGGTTTGATCCCAGC-3’; for *PLCL1* (At1g13680), 5’- CCGTCAACTTTTACAAGAGG-3’ and 5’-AGCATGCACATCATCACGTC-3’; for *DORN1* (At5g60300), 5’-TGCAGTTGACAAATGCTTCAG-3’ and 5’-TTCAGACATCTCATGCTCACG-3’; for *ACTIN-2* (At3g18780), 5’-GGATCGGTGGTTCCATTCTTGC-3’ and 5’-AGAGTTTGTCACACACAAGTGCA-3’; *EIF4A* (At3g13920), 5’-ATGAGAGGATGCTCTGCCTTCG-3’ and 5’-GCAGAGCAAACACAGCAACAG-3’.

Statistical analyses

Gene expression and quantitative cell death assay results were analysed using ANOVA and Tukey-Kramer post hoc test.

References

Chivasa S, Ndimba BK, Simon WJ, Lindsey K, Slabas AR. 2005. Extracellular ATP functions as an endogenous external metabolite regulating plant cell viability. *Plant Cell* **17:** 3019–3034.
